# Synthetic feed-forward loop circuit boosts transgene expression in sugarcane

**DOI:** 10.1007/s00299-025-03561-3

**Published:** 2025-07-08

**Authors:** Zhihai Zhang, Sultana Anwar, Erin J. Yafuso, Evelyn Tatiana Zuniga Soto, Changwei Li, Guangbin Luo, Stephen P. Moose, Kankshita Swaminathan, Fredy Altpeter, Matthew E. Hudson

**Affiliations:** 1 DOE Center for Advanced Bioenergy and Bioproducts Innovation (CABBI), University of Illinois at Urbana-Champaign, Urbana, IL 61801 USA; 2 Department of Crop Sciences, University of Illinois at Urbana-Champaign, 1102S Goodwin Ave, Urbana, IL 61801 USA; 3 Agronomy Department, University of Florida, 3105 McCarty Hall B, Gainesville, FL 32603 USA; 4 Center for Advanced Bioenergy and Bioproducts Innovation, University of Florida, 3105 McCarty Hall B, Gainesville, FL 32603 USA; 5 HudsonAlpha Institute for Biotechnology, Huntsville, AL 35806 USA

**Keywords:** Global sustainability, Biobased products, Feed-forward loop circuit, GAL4 system, Sugarcane

## Abstract

A new GAL4-based feed-forward loop circuit enhances β-glucuronidase (GUS) reporter gene expression in leaves and stems of stably transformed sugarcane plants.

Bio-based products derived from natural plant-derived materials offer a promising alternative to petroleum-based products, which are essential for achieving global sustainability (Gupta et al. 2022). Sugarcane provides 40% of the world’s biofuel (Brant et al. 2025). Recently, metabolic engineering of sugarcane for hyperaccumulation of biomass oil is emerging as a strategy to elevate the crop’s energy content (Cao et al. 2023; Maitra et al. 2024). Synthetic transcription factors offer a powerful tool for modulating entire metabolic pathways by enabling fine-tuned activation or repression of specific genes (Liu and Stewart 2016; Hooghvorst and Altpeter 2023). However, the application of these technologies faces challenges, including the availability of well-characterized genetic building blocks for precise manipulation of gene expression, and the inherently challenging properties of gene expression in highly polyploid crops like sugarcane (Liu and Stewart 2016).

In this study, a version of the GAL4 system, originally developed for enhancer trap assays in Arabidopsis (Engineer et al. 2005), and the EDLL transcription activation domain (Tiwari et al. 2012) were combined with the promoter of the *Dry* locus in sorghum (Fujimoto et al. 2018), *pSbDry*, to control the expression of the GUS reporter gene as a coherent feed-forward loop circuit, *pBEC64*, as shown in Fig. 1A. A control construct, *pBEC16*, was built to determine the reporter gene expression level under the *pSbDry* promoter in the absence of the GAL4-binding domain and EDLL transcription activation domain (Fig. 1B). An additional control vector, *pALS1*, was constructed like *pBEC64* except that the GUS reporter gene was under transcriptional control of a weak constitutive promoter from the maize *acetolactate synthase 1* (*ALS1*) gene, lacking GAL4-binding sites (Fig. 1C). Biolistic gene transfer was used to introduce the constructs into sugarcane embryonic calli, derived from sugarcane leaf whorls of cultivar CP88-1762. Histochemical GUS staining was conducted 2 days after biolistic gene transfer using both its linear and circular cassettes, with *pALS1* control vector as a reference (Fig. 1D). Compared with the *pALS1* control, both linear and circular *pBEC64* exhibited stronger GUS expression in callus, suggesting that the synthetic feed-forward loop construct may be effective at enhancing expression in sugarcane callus.

**Fig. 1:**
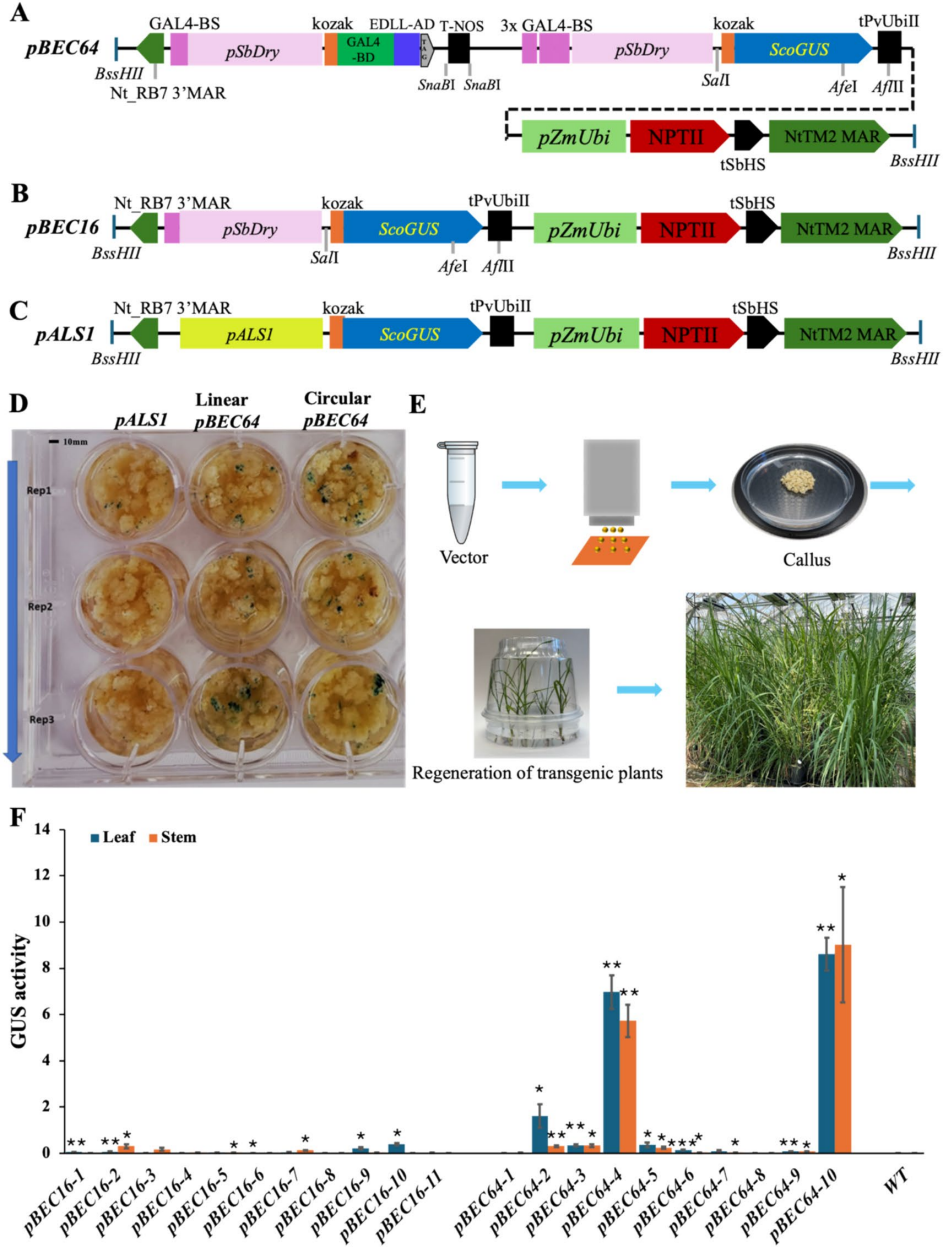
Evaluation of the synthetic feed-forward loop circuit in sugarcane. **A** Functional feed-forward loop, *pBEC64*; *BD* binding domain, *AD* activation domain, *BS* binding site. The anticipated operation mechanism of the coherent feed-forward loop circuit is: 1, the *pSbDry* promotes the low-level constitutive expression of GAL4-BD::EDLL-AD; 2, the GAL4-BD::EDLL-AD product binds to GAL4-BS; 3, and activates the *pSbDry* promoter to elevate the expression of GAL4-BD::EDLL-AD; 4, the GAL4-BD::EDLL-AD also binds to the 3 × GAL4-BS; 5, and also actives the promoter *pSbDry*; 6, highly activated *pSbDry* promotes the expression of the *ScoGUS* reporter. **B** *pBEC16*, with GAL-BS::*pDry*::kozak::*ScoGUS* and without the GAL4-BD and DELL-AD used as control. **C** *pALS1*, *ScoGUS* reporter is driven by a weak constitutive promoter from the maize *acetolactate synthase 1* (*ALS1*) gene. **D** Transient gusA reporter gene expression assay. The linearized and circular plasmids of the *pBEC64* vector were both evaluated with histochemical GUS staining 2 days after biolistic gene transfer into callus. Marked GUS staining intensity was observed compared with the control vector (*pALS1*). **E** Generation of transgenic sugarcane. Vectors were bombarded into embryogenic sugarcane callus. Transgenic calli were selected with geneticin-containing culture medium and regenerated into plants. Transgenic plants were grown to maturity in the greenhouse. **F**, Quantitative evaluation of GUS activity via MUG assay from stem and leaf tissue of stable transformed plants harboring *pBEC64* or *pBEC16* constructs compared with wild-type sugarcane (WT) (*n* = 3). *p < 0.05, **p < 0.01 ≤ *p* < 0.05, ***p < 0.01

*pBEC64* and *pBEC16* were compared in stably transformed sugarcane plants following selection and regeneration on geneticin containing culture medium (Fig. 1E). Ten and eleven independent transgenic events for *pBEC64* and *pBEC16,* respectively, were evaluated for quantitative GUS reporter gene expression using MUG assays following protein normalization with the Bradford assay, using Quick Start™ Bradford dye reagent from Bio-Rad (Hercules, CA) and Multi-Mode Microplate SpectraMax M5 fluorometer from Molecular Devices (San Jose, CA) at 595 nm absorbance. Each transgenic event had 3–5 plants. Three biological replicates and three technical replicates were analyzed for both leaf and stem samples from each transgenic event at the tillering stage, characterized by the 3–4 emerging internodes. The middle section of the stems and the first dewlap leaves were sampled. 4-methylumbelliferyl-β-D-glucuronide hydrate (4-MUG) from GoldBio (St. Louis, MO) Co. and 4-methylumbelliferone (4-MU) from Sigma-Aldrich (St. Louis, MO) were used for the MUG assay. The amount of 4-MU produced from 4-MUG upon GUS hydrolysis was quantified by the standard curve calibrated from 4-MU concentration gradient: 1000, 500, 150, 50, and 20 nM, using the Multi-Mode Microplate Reader SpectraMax M5 with 365 nm excitation and 455 nm emission filter. All quantified GUS activities in transgenic tissues were statistically compared with those in wild type.

Among the transgenic events, 45.5% (5 /11) and 27.3% (3/11) of *pBEC16*, and 70.0% (7/10) and 80.0% (8/10) of *pBEC64* showed significantly higher GUS activities than the wild type (CK) in leaf and stem tissues, respectively (Fig. 1F). The *pBEC64-10* showed the highest overall GUS activity in leaves with a GUS activity of 8.36 nmoles MU/min/mg protein, and stems with a GUS activity of 9.02 nmoles MU/min/mg protein. The *pBEC64-4* showed the second highest GUS activity in leaves with a GUS activity of 6.98 nmoles MU/min/mg protein, and stems with a GUS activity of 5.74 nmoles MU/min/mg protein. In addition, the *pBEC64-2* exhibited significant stem-specific expression, although the GUS activities in both stems and leaves were significantly higher compared with the wild type (WT).

The 11 *pBEC16* control events displayed no or low-level constitutive GUS expression (Fig. 1F) in contrast to the high-level GUS expression of several *pBEC64* events. These findings provide evidence that the GAL4-BD::EDLL artificial transcription factor is functional, and that the feed-forward loop construct (*pBEC64*) is effective at enhancing gene expression in sugarcane.

Synthetic biology shows great promise for the rational design of genetic circuits to confer expression to genes or molecular pathways of interest (Liu & Stewart 2016). Modulating transgene expression with the help of heterologous transcription factors and promoters can be challenging (Yaschenko et al. 2022) in complex polyploid genomes like sugarcane.

In our study, the synthetic feed-forward construct *pBEC64* enabled strong upregulation of the weak *pSbDry* promoter in both stem and leaf tissues. Elevating transgene expression in sugarcane has great potential for pathway engineering to fuel the emerging bioeconomy (Cao et al. 2023; Maitra et al. 2024).

In conclusion, we designed and built a coherent feed-forward loop circuit for plants and evaluated its performance in sugarcane calli, leaves, and stems, demonstrating the circuit's effectiveness in enhancing stable and robust transgene expression in sugarcane, with potential broader applications in plant synthetic biology. Future applications could involve adapting this circuit to regulate different target genes involved in metabolic engineering, stress resilience, or developmental pathways, and expanding its use to other crops by customizing the promoter or transcriptional activator components to match species-specific expression profiles.
